# Expression, Purification, and Anti-UV Irradiation Effect of RsSOD on HCE-T Human Corneal Epithelial Cells

**DOI:** 10.3390/genes15091147

**Published:** 2024-08-30

**Authors:** Xucong Fu, Zhuo Jiang, Wenhui Bi, Zhecheng Yang, Weina Lu, Jianqing Chen, Zhengbing Lyu, Zuoming Nie

**Affiliations:** College of Life Sciences and Medicine, Zhejiang Sci-Tech University, Hangzhou 310018, China; 13082839386@163.com (X.F.); jzhuo2022@163.com (Z.J.); q17857027985@163.com (W.B.); most.authentic.self@gmail.com (Z.Y.); lwn2421081852@163.com (W.L.); cjqgqj@zstu.edu.cn (J.C.); zhengbingl@zstu.edu.cn (Z.L.)

**Keywords:** superoxide dismutase, stability, HCE-T cells, ultraviolet irradiation, anti-irradiation effect

## Abstract

Superoxide dismutase (SOD) is a class of enzymes that catalyze the disproportionation of superoxide anion radicals into hydrogen peroxide and oxygen. It can remove excessive free radicals in organisms and acts as a potent antioxidant, cleaning free radicals generated by radiation and protecting cells from oxidative damage. In this study, we obtained a MnSOD gene from the radiation-resistant bacterium *Radiobacillus* sp. (RsSOD) and constructed its recombinant expression vector through gene synthesis. The recombinant RsSOD protein was efficiently expressed using IPTG induction, and purified via repeated freezing and thawing, heating, and DEAE anion-exchange chromatography. The purified RsSOD exhibited an enzyme activity of 2072.5 U/mg. Furthermore, RsSOD was demonstrated to have robust resistance to high temperatures, acid, alkali, and artificial intestinal fluid. Further studies were performed to investigate the radiation resistance of RsSOD against ultraviolet (UV) irradiation in human corneal epithelial (HCE-T) cells. The results indicated that a low concentration of RsSOD (6.25 U/mL) could promote HCE-T cell proliferation and protect these cells from damage caused by both long-term and short-term UV exposure, effectively reducing apoptosis induced by short-term UV irradiation. These findings suggest that the RsSOD protein possesses significant anti-UV irradiation property and is expected to be a candidate for treating ocular radiation-related diseases.

## 1. Introduction

Superoxide dismutase (SOD) is a class of catalytic enzymes that convert superoxide into oxygen and hydrogen peroxide via a disproportionation reaction, as follows:

SOD + 2O_2_^−^ + 2H^+^→SOD + H_2_O_2_ + O_2_, where O_2_^−^ is the superoxide anion, and H^+^ denotes a proton. Superoxide anions are one of the most prevalent free radicals produced during cellular metabolism. They can cause oxidative damage to cells, resulting in various adverse effects and diseases. SOD can effectively remove superoxide anion free radicals generated within cells, thereby reducing oxidative stress damage to cell membranes, proteins and DNA, and protecting cells from oxidative damage [1]. SOD is divided into four types, Cu/ZnSOD, MnSOD, FeSOD, and NiSOD [2]. SOD has great medical value as an important antioxidant enzyme and plays a key role in the treatment of many diseases, such as skin damage [3], brain diseases [4], inflammatory eye diseases [5,6], and meconium aspiration syndrome [7]. In the future, the medical value of SOD will continue to be developed [1].

There is a strong association between ultraviolet (UV) exposure and ocular diseases, making it a key factor in causing or aggravating some ocular diseases, such as pterygium, opacities, and cataracts [8]. When the eye is exposed to UV light, it results in an increase in reactive oxygen species (ROS) and induces oxidative stress. This process directly damages proteins and cellular structures in the eye, thereby adversely impacting ocular health [9]. The cornea, as the transparent outer tissue of the eye and the first line of defense, becomes the primary target of UV damage [10]. This damage can be severe, potentially leading to the loss of keratocytes in the central cornea and thereby triggering various ocular diseases associated with UV irradiation [11,12].

MnSOD, an essential antioxidant enzyme, can efficiently clear superoxide anions, thereby significantly alleviating the cell and tissue damage induced by ionizing radiation [13,14,15,16]. Previous research has demonstrated the protective effects of SOD against oxidative damage and ocular diseases. Exogenous SOD has been shown to decrease malondialdehyde (MDA) levels in rabbit retinal tissue and increase SOD activity; thus, the increased SOD can effectively clear the free radicals to protect the cell membrane from oxidant damage in the retina [17]. Another study indicated that MnSOD provides substantial antioxidant protection to lens epithelial cells [13]. This protective mechanism involves the reduction of intracellular ROS accumulation, thereby shielding cells from oxidative stress [18]. Further study has also confirmed that MnSOD is associated with the development of radiation-induced cataracts in mice [19], and that the addition of MnSOD significantly reduces the lens opacity of eyes in UV-irradiated animals. This may be due to the fact that MnSOD blocked the pathogenic pathway of irradiation-induced lens opacity formation or that MnSOD modulation reduced the effects of UV exposure [20]. In addition, the human recombinant MnSOD was also determined to treat photoaging and actinic keratosis [21]. Collectively, these studies show the potential therapeutic application of MnSOD in treating UV-radiation-induced ocular diseases.

In this study, we obtained a gene encoding the MnSOD protein from an irradiation-resistant bacillus *Radiobacillus kanasensis*, named RsSOD. Subsequently, the RsSOD protein was expressed in *Escherichia coli*, purified, and evaluated for stability against various conditions including heat, acid, alkali, and artificial intestinal fluid. Additionally, we investigated the protective and repairing effects of the RsSOD protein on UV-induced damage in HCE-T human corneal epithelial cells. This research aims to propose a novel strategy for preventing and treating UV-induced damage to corneal epithelial cells, thereby offering a theoretical foundation for the development of new anti-photoaging therapies.

## 2. Materials and Methods

### 2.1. Strain and Cell Line

The *E. coli* BL21 (DE3) strain was kept in our laboratory. The human corneal epithelial cell line HCE-T was obtained from RIkagaku KENkyusho and cultured using DMEM/F-12 complete medium, which consisted of 15% fetal bovine serum (FBS), 100 U/mL penicillin, 0.1 mg/mL streptomycin, 10 ng/mL epidermal growth factor (EGF), and 5 μg/mL insulin.

### 2.2. Bioinformatics Analysis

The genome of irradiation-resistant Bacillus *R. kanasensis* was accessed from the NCBI database (Accession no. GCF_021049245.1). A potential *MnSOD* gene, named *RsSOD*, was identified from this dataset. Orthologs of RsSOD were retrieved using the Blastp program available at NCBI. The tertiary structure of RsSOD was predicted using RoseTTAfold, accessible at http://robetta.bakerlab.org (accessed on 4 April 2024), and subsequently visualized with PyMol (https://www.pymol.org/, accessed on 26 August 2024).

### 2.3. Expression and Purification of RsSOD Protein

The ORF sequence of the *RsSOD* gene was synthesized by Suzhou GENEWIZ Biotechnology Co., Ltd (Suzhou, China). and subsequently cloned into the pET-28a(+) vector to create the recombinant prokaryotic expression vector pET-28a(+)-RsSOD. The expression of the recombinant RsSOD protein was induced with 1 mM IPTG (Isopropyl β-D-1-thiogalactopyranoside) at 30 °C and 220 rpm for 8 h in *E. coli* BL21(DE3). The bacterial culture was then centrifuged at 12,000 rpm for 1 min and subjected to ultrasonic disruption. Following ultrasonication, centrifugation at 12,000 rpm for 3 min separated the supernatant and pellet. SDS-PAGE analysis was performed to confirm the expression and expression pattern of RsSOD.

Subsequently, freeze-thaw cycles and heat treatment were employed for initial RsSOD purification. Firstly, the RsSOD recombinant bacteria underwent five freeze-thaw cycles alternately at −80 °C and room temperature. Following freeze-thawing, an equal volume of buffer A (20 mM Tris-HCl, pH 8.03) was added, followed by centrifugation at 10,000 rpm for 5 min. The resulting supernatant was heated in a water bath at temperatures of 60 °C, 70 °C, 80 °C, and 90 °C for 10 min, respectively, followed by centrifugation at 12,000 rpm for 10 min. The RsSOD protein in the supernatant was further purified using DEAE (Diethylaminoethyl) anion-exchange chromatography using a linear gradient elution employing 5 column volumes (CV) of buffer B (1.0 M NaCl solution).

### 2.4. Stability Determination of RsSOD Protein

Firstly, the thermal stability of RsSOD was determined. The protein was heated in a water bath at 37 °C, 50 °C, 60 °C, 80 °C, and 90 °C for 30 min, respectively, and then the enzyme activity was determined using the Marklund method (Chinese national standard: GB/T5009.171, accessible at https://openstd.samr.gov.cn/bzgk/gb, accessed on 4 April 2024). The enzyme activity at 37 °C was set as 100%, and the relative enzyme activity at the different temperatures was calculated. Secondly, the pH stability of RsSOD was further determined. The protein was added into the different buffers of pH 2.0~12.0, respectively, and placed in a water bath at 37 °C for 30 min. The enzyme activity at pH 7.0 was set as 100%, and the relative enzyme activity at each different pH was calculated. Finally, the stability of RsSOD treated with artificial intestinal fluid was determined. Artificial intestinal fluid is a simulated intestinal fluid containing pancreatic enzymes (pH = 6.8 ± 0.1) and purchased from Guangjian Testing Technology Co., Ltd (Guangzhou, China). The protein was mixed with the artificial intestinal fluid at 37 °C for 0 min, 20 min, 40 min, 60 min, 80 min, 100 min, and 120 min, respectively. The enzyme activity at 0 min was set as 100%, and the relative enzyme activity at the different times was calculated.

### 2.5. Effect of RsSOD Protein on the Proliferation of HCE-T Cells

HCE-T cells were cultured to the logarithmic growth phase, placed onto 96-well plates at 3000 cells/well, and then the cells were cultured at 37 °C and 5% CO_2_. After 24 h, when the cells were fully adherent to the wall and grew to about 80%, the medium was replaced with fresh media containing serial concentrations of RsSOD as follows: 3.125 U/mL, 6.25 U/mL, 12.5 U/mL, 25 U/mL, 50 U/mL, 100 U/mL, 200 U/mL, 400 U/mL, and 800 U/mL, respectively. After culturing for 24 h, the cell viability was assessed by the CCK-8 method. The CCK-8 method, also known as the Cell Counting Kit-8, is widely used for the rapid and highly sensitive detection of cell proliferation and cytotoxicity. This method is based on the reducibility of WST-8 solution. Briefly, the old medium was discarded, and the mixture of fresh medium and CCK-8 solution was added into the wells. After the cells were incubated for 1 h, the absorbance at 450 nm was detected by a microplate absorbance reader, and the cell viability was then calculated. The group with 0 U/mL was used as the control group.

### 2.6. Effect of Long-Term or Short-Term UV Irradiation on the Proliferation of HCE-T Cells

HCE-T cells were cultured as above. When the cells were fully adherent to the wall and grew to about 80%, the 96-well plates were placed directly under UV light at a vertical distance of 7 cm and then irradiated. UV light from Shanghai Lichen Instrument Technology (Model ZF-1) was employed, with the ultraviolet intensity set at 50 V ± 10 V. For the long-term UV irradiation, UV light with wavelengths of 365 nm and 254 nm was selected to irradiate the cells for 10 min and 15 min, respectively. For the short-term UV irradiation, UV light with a wavelength of 254 nm was selected to irradiate the cells for 1 min, 3 min, and 5 min, respectively. After irradiation, the cells were cultured continuously in the cell culture incubator for 48 h. After culturing, the cell viability was assessed by the CCK-8 method to select the appropriate radiation time for the long-term or short-term UV irradiation. The group with no UV irradiation was used as the control group.

### 2.7. Effect of RsSOD on the Proliferation of HCE-T Cells under UV Irradiation

To determine the optimal dose of RsSOD against UV irradiation in HCE-T cells, the cells were treated with serial RsSOD concentrations of 0 U/mL, 3.125 U/mL, 6.25 U/mL, 12.5 U/mL, 25 U/mL, 50 U/mL, and 100 U/mL, respectively, and then exposed to the same long-term UV irradiation as mentioned above. After irradiation, the cells were continuously cultured for 0 h and 2 h, respectively, and the cell viability was assessed by the CCK-8 method to determine the optimal dose of RsSOD. When the optimal dose of RsSOD was determined, the groups treated with 0 U/mL and the optimal dose of RsSOD were continuously cultured for 8 h, 24 h, and 36 h, respectively. The growth status of the cell morphology and the number of cells were observed using an inverted microscope. After observation, the cell viability was also assessed by the CCK-8 method to identify the effect of the optimal dose of RsSOD on the proliferation of HCE-T cells under long-term UV irradiation. The 0 U/mL group without UV irradiation for each time was used as the control group.

Furthermore, to identify the effect of RsSOD on the proliferation of HCE-T cells under short-term UV irradiation, the cells were treated with 0 U/mL and the optimal dose of RsSOD, and then exposed to the same short-term UV irradiation mentioned above. When the cells were continuously cultured for 0.5 h, 5 h, 12 h, and 24 h, respectively, the growth status of the cell morphology and the number of cells were observed using an inverted microscope. After observation, the cell viability was also assessed by the CCK-8 method to identify the effect. The 0 U/mL group without UV irradiation for each time was used as the control group.

The apoptosis of the cells cultured for 24 h was further analyzed by a flow cytometer. The cell culture medium was collected, and 500 μL of 0.25% trypsin was added to each well to dissociate the adherent cells. The cell mixture collected above was centrifuged at 800 rpm for 3 min to collect the cell pellet. The cell pellet was washed with 1 mL of pre-cooled PBS and resuspended with 300 μL of 1 × Binding Buffer. Then, different reagents were added for different groups, as follows: Control group 1: no addition; control group 2: 3 μL Annexin V-FITC solution; control group 3: 3 μL PI solution; experimental group: 3 μL Annexin V-FITC and 3 μL PI. Each group was mixed and kept in the dark for 15 min, and then 300 μL of 1 × Binding Buffer was added. After mixing, the apoptosis analysis was performed by flow cytometry.

### 2.8. Statistical Analysis

Data and histograms were analyzed for significance using GraphPad Prism software (version 8.0). Each group contained 6 parallel replicated samples. The differences between the groups were assessed by a t-test (*n* ≥ 3). Statistical significance was expressed as * *p* < 0.05, ** *p* < 0.01, *** *p* < 0.001, and **** *p* < 0.0001 for difference, significant difference, highly significant difference, and very highly significant difference, respectively.

## 3. Results

### 3.1. Bioinformatics Analysis

**Figure 1:**
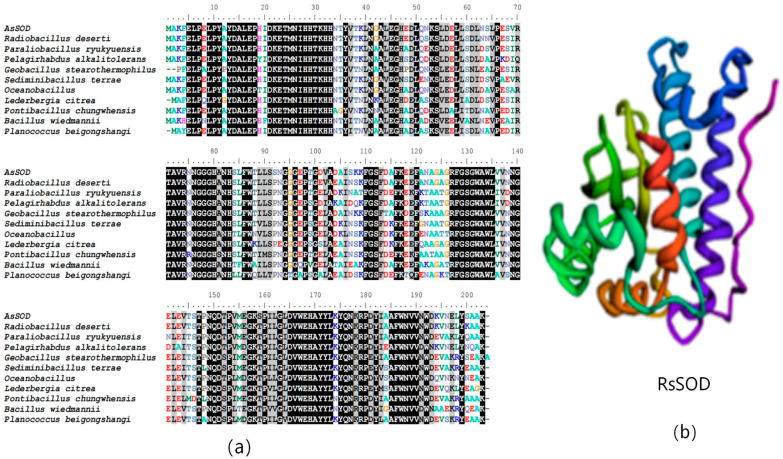
Bioinformatics analysis of RsSOD. (**a**) Multiple sequence alignment of SOD proteins. (**b**) Tertiary structure prediction of RsSOD.

The open reading frame (ORF) of the RsSOD gene (GCF_021049245.1: 2153884-2154495) was 612 bp in length, encoding 203 amino acid residues, with a calculated molecular weight of 22.6 kDa. The Blastp analysis identified 10 orthologs, and the subsequent multiple sequence alignment indicated the high conservation of the RsSOD protein at the N- and C-termini, as well as in the central region, which is likely associated with its enzyme activity (Figure 1a). Tertiary structure prediction using RoseTTAfold revealed that RsSOD predominantly comprises α-helices, with three small β-sheets in the middle, suggesting a stable tertiary structure (Figure 1b). Consequently, RsSOD is predicted to possess thermal stability.

### 3.2. Expression and Purification of RsSOD Protein

**Figure 2:**
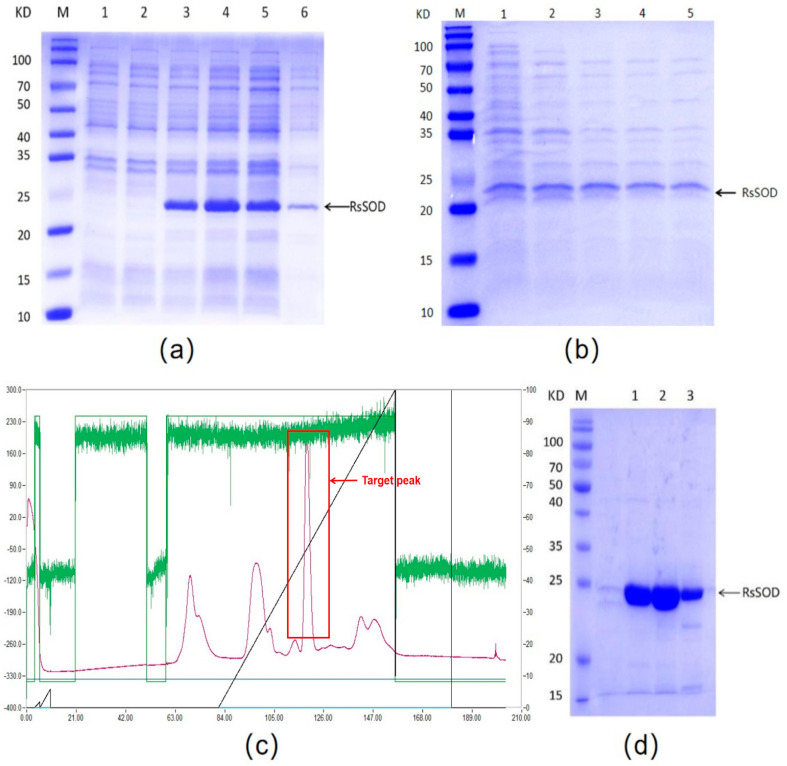
Expression and purification of the recombinant RsSOD protein. (**a**) Prokaryotic expression of recombinant RsSOD. M: pre-stained protein marker; 1: uninduced bacteria harboring pET−28a(+) vector; 2: uninduced RsSOD engineered bacteria; 3: induced RsSOD engineered bacteria; 4, 5: supernatant after ultrasonication; 6: pellet after ultrasonication. (**b**) Removal of thermolabile proteins. 1: no heating; 2, 3, 4, 5: heating at 60 °C, 70 °C, 80 °C, 90 °C, respectively. (**c**) Chromatogram of DEAE anion-exchange chromatography. (**d**) SDS-PAGE analysis for the target peak. 1, 2, 3: eluent of the target peak.

IPTG was employed to induce the expression of RsSOD in *E. coli* BL21(DE3). The induced recombinant bacteria harbored a distinct band (22.6 kDa) matching the molecular weight of RsSOD, in contrast to the uninduced control. Moreover, the majority of the recombinant RsSOD protein was found in the supernatant following ultrasonic disruption (Figure 2a).

The expressed RsSOD was subsequently purified. Initially, the recombinant bacteria were lysed via freeze-thaw cycles, and the resulting supernatant after centrifugation was heat-treated to remove other thermolabile proteins based on the thermal stability of the RsSOD protein. It was observed that heating at 70 °C effectively eliminated most of these proteins (Figure 2b), thereby enabling the preliminary purification of RsSOD. Furthermore, even when the heating temperature was raised to 90 °C, the RsSOD protein also survived, suggesting that RsSOD should be heat-resistant. Subsequently, the RsSOD protein was further purified via DEAE anion-exchange chromatography using a gradient elution with 1 M NaCl. As illustrated in Figure 2c, a prominent peak emerged within the salt concentration range of 450 mM to 530 mM. SDS-PAGE analysis confirmed that the highly purified RsSOD protein could be obtained through the combination of heat treatment and anion-exchange chromatography (Figure 2d). Based on the total enzyme activities of both the pre-purified and purified protein solutions, the purification yield efficiency was calculated to be approximately 85%.

### 3.3. Stability of RsSOD Protein

**Figure 3:**
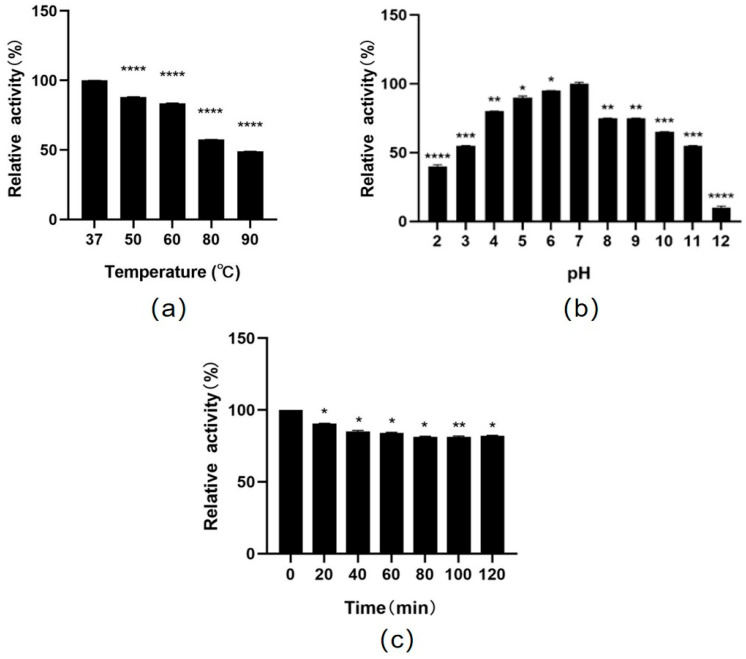
Assessment of the stability of the RsSOD protein. (**a**) Enzyme activity of RsSOD at different temperatures. (**b**) Enzyme activity of RsSOD at different pH. (**c**) Enzyme activity of RsSOD treated with artificial intestinal fluid at different times. * *p* < 0.05, ** *p* < 0.01, *** *p* < 0.001, and **** *p* < 0.0001.

The enzyme activity of the purified RsSOD protein was measured to be 2072.5 U/mg, and its stability was subsequently evaluated, including resistance to heat, acid, alkali, and artificial intestinal fluid. RsSOD retained most of its enzyme activity after treatment at different high temperatures. Notably, it retained approximately 49% of its activity after treatment at 90 °C for 30 min, demonstrating robust thermal stability (Figure 3a). Moreover, compared to its activity at pH 7.0, RsSOD retained more than 50% of its enzyme activity across a wide pH range from 3.0 to 11.0, suggesting strong acid and alkali resistance (Figure 3b). Lastly, the impact of artificial intestinal fluid on RsSOD enzyme activity was assessed. As shown in Figure 3c, RsSOD exhibited a low loss of enzyme activity when exposed to artificial intestinal fluid, indicating its excellent tolerance to artificial intestinal fluid.

### 3.4. Determination of Optimal Dose of RsSOD against UV Irradiation on HCE-T Cells

**Figure 4:**
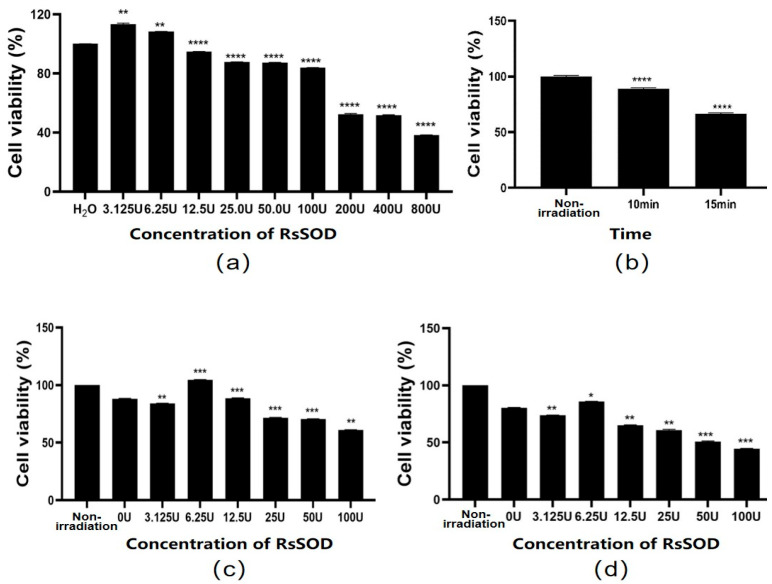
Determination of the optimal dose of RsSOD against UV irradiation on HCE-T cells. (**a**) Effect of RsSOD on the proliferation of HCE-T cells. The RsSOD concentrations are written on the horizontal axis. (**b**) The survival rate of HCE-T cells after different time periods of UV irradiation. The cell viability of UV-irradiated HCE-T cells treated with different concentrations of RsSOD after (**c**) 0 h (**d**) and 2 h of incubation, respectively. * *p* < 0.05, ** *p* < 0.01, *** *p* < 0.001, and **** *p* < 0.0001.

The anti-irradiation effect of RsSOD on HCE-T cells under UV exposure was further investigated. Firstly, the effect of RsSOD on the proliferation of HCE-T cells was assessed. It was found that lower concentrations of RsSOD, specifically 3.125 U/mL and 6.25 U/mL, promoted the proliferation of HCE-T cells. In contrast, concentrations exceeding 12.5 U/mL inhibited HCE-T cell proliferation, particularly the concentration exceeding 200 U/mL that resulted in cell damage exceeding 50% (Figure 4a). Therefore, these high concentrations of SOD were unsuitable for use in anti-irradiation therapy. Next, the damage of long-term UV irradiation on HCE-T cells was evaluated. After exposure to UV irradiation at 365 nm and 254 nm for 10 min, cell viability decreased by approximately 12%. This reduction increased to around 30% after 15 min of UV exposure, which was selected for investigating the protective effect of RsSOD against UV irradiation (Figure 4b). Subsequently, cells were exposed to UV irradiation for 15 min following the addition of RsSOD and were cultured for 0 h and 2 h, respectively. A comparative analysis revealed that RsSOD at 6.25 U/mL significantly enhanced cell viability, suggesting an optimal dose of RsSOD against UV irradiation on HCE-T cells (Figure 4c,d).

### 3.5. Effect of RsSOD against Long-Term UV Irradiation in HCE-T Cells

**Figure 5:**
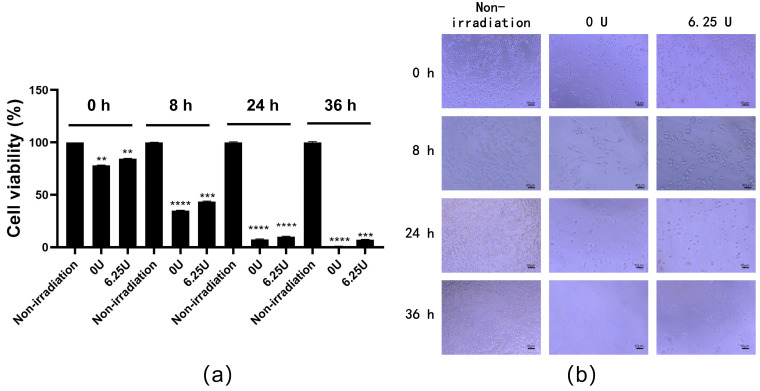
Effect of RsSOD with a concentration of 6.25 U/mL on the proliferation of long-term UV-irradiated HCE-T cells. (**a**) Viability of HCE-T cells; (**b**) morphology of HCE-T cells. ** *p* < 0.01, *** *p* < 0.001, and **** *p* < 0.0001.

The HCE-T cells irradiated with UV as described above were treated with 6.25 U/mL RsSOD and subsequently cultured for 8 h, 24 h, and 36 h, respectively. Over time, a marked decrease in cell viability was observed. Nevertheless, cells treated with RsSOD exhibited significantly higher viability compared to untreated cells (Figure 5a). An analysis of cell morphology revealed that, following long-term UV irradiation, cell boundaries became indistinct and cell numbers decreased, indicating severe cellular damage due to UV exposure. However, irradiated cells treated with RsSOD showed fewer alterations in morphology compared to irradiated cells untreated with RsSOD (Figure 5b), suggesting that RsSOD at 6.25 U/mL has a better anti-UV irradiation effect on HCE-T cells.

### 3.6. Effect of RsSOD against Short-Term UV Irradiation in HCE-T Cells

**Figure 6:**
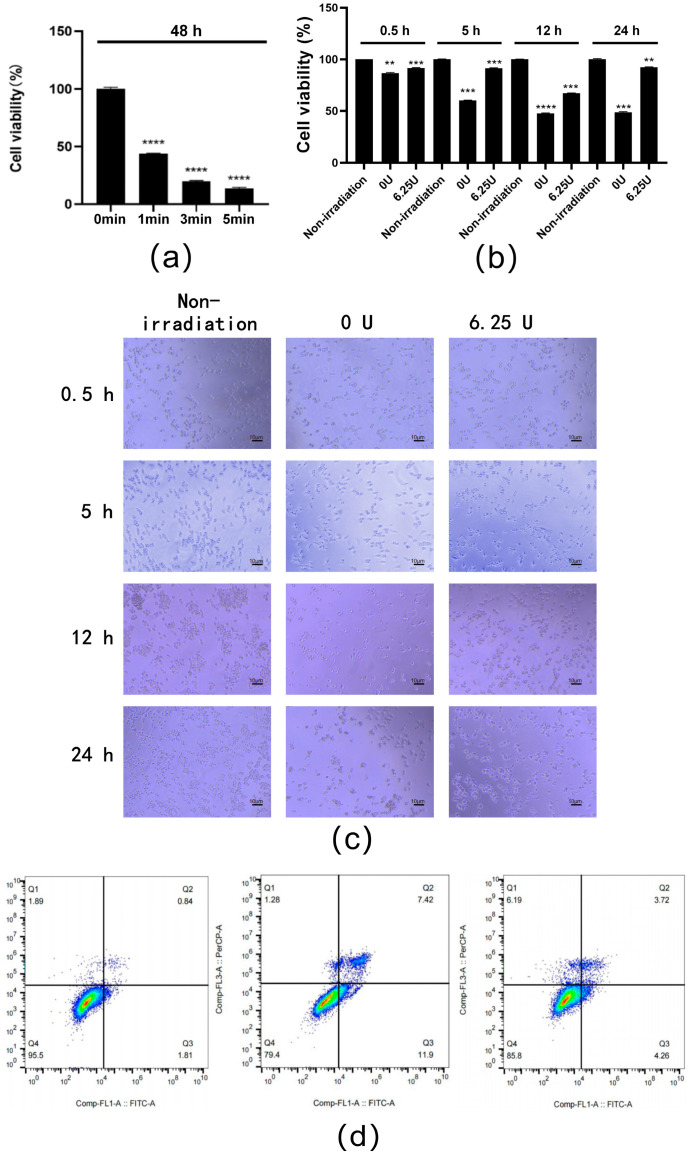
Effect of RsSOD treatment on short-term UV-irradiated HCE-T cells. (**a**) Viability of cells under different times of UV exposure. The cells were cultured for 48 h. (**b**) Effect of RsSOD treatment on the proliferation of short-term UV-irradiated cells. (**c**) Morphology of cells. (**d**) Effect of RsSOD treatment on apoptosis of short-term UV-irradiated cells. Left: non-irradiated cells untreated with RsSOD; middle: short-term UV-irradiated cells untreated with RsSOD; right: short-term UV-irradiated cells treated with RsSOD. The number of cells used for each experiment is 10,000, and there are three experimental repeats for each sample. ** *p* < 0.01, *** *p* < 0.001, and **** *p* < 0.0001.

To investigate the anti-irradiation effect of the RsSOD protein, the proliferation of HCE-T cells under short-term UV irradiation was examined. The results demonstrated that exposure to 254 nm UV light for only 1 min reduced cell viability by 60% and was then used for further investigation into the protective effects of RsSOD (Figure 6a). As the incubation time increased, the viability of short-term UV-irradiated cells progressively decreased; however, treatment with 6.25 U/mL of RsSOD significantly improved viability, which was particularly evident at 24 h post-exposure, where RsSOD increased viability by 90.4% (Figure 6b). Additionally, the RsSOD-treated UV-irradiated cells exhibited a better cell growth status and increased cell numbers (Figure 6c), indicating a repairing effect of 6.25 U/mL RsSOD on HCE-T cells damaged by short-term UV exposure.

Flow cytometry analysis at 24 h post-culture further revealed that short-term UV irradiation increased apoptosis by 16.67% in cells untreated with RsSOD, suggesting a pro-apoptotic effect. Meanwhile, treatment with 6.25 U/mL RsSOD reduced apoptosis by 11.34% in UV-irradiated cells (Figure 6d), indicating a protective role of RsSOD against UV-induced apoptosis. These findings collectively demonstrate that 6.25 U/mL RsSOD could possess anti-UV irradiation properties, mitigating the cell damage and apoptosis induced by short-term UV exposure, potentially offering therapeutic benefits for corneal cell damage caused by low-dose UV irradiation.

## 4. Discussion

UV irradiation can lead to the production of a large amount of ROS, such as superoxide anions, resulting in oxidative stress in cells, which, in turn, causes cellular damage [22,23]. As a kind of SOD, MnSOD can eliminate ROS via a disproportionation reaction; therefore, MnSOD is a potential candidate for treating UV-irradiation-induced ocular diseases by mitigating the oxidative stress damage caused by UV exposure. Studies indicated that MnSOD can prevent Polγ inactivation and protect mitochondrial DNA (mtDNA) from UV-induced oxidative damage. MnSOD interacts with mtDNA, Polγ, and glutathione peroxidase, collectively acting as an antioxidant [24,25,26]. Thus, it is positively significant to investigate the protective role of MnSOD protein against UV irradiation in HCE-T cells. 

In this study, an RsSOD gene encoding the MnSOD protein was screened from the genome of radiation-resistant Bacillus and subsequently expressed efficiently in prokaryotes. The RsSOD protein was purified to high purity using a two-step procedure involving heat treatment and anion-exchange chromatography, achieving a specific enzyme activity of 2072.5 U/mg. Compared to the previous traditional method for SOD protein purification including ammonium sulfate precipitation [27], this simplified two-step method is more suitable for the large-scale preparation of high-purity SOD proteins. Protein stability assays demonstrated that RsSOD exhibits robust resistance to high temperatures, acidic and alkaline conditions, and some tolerance to artificial intestinal fluids, suggesting broad potential applications due to its stability. 

The radiation resistance observed in certain bacteria might be attributed to the anti-irradiation effect of their biological macromolecules, such as proteins. Therefore, RsSOD, derived from the radiation-resistant Bacillus *R. kanasensis*, could exhibit better anti-irradiation effects compared to SOD derived from non-radioresistant bacteria. Further investigations into the anti-irradiation effect of the RsSOD protein on HCE-T cells were conducted. The results revealed that low concentrations of RsSOD (3.125 U/mL and 6.25 U/mL) could promote the proliferation of HCE-T cells. Notably, 6.25 U/mL of RsSOD demonstrated protective effects against both long-term and short-term UV irradiation-induced damage to HCE-T cells, reducing the apoptosis caused by short-term UV exposure. These findings indicated that an appropriate concentration of RsSOD has effectiveness against UV irradiation; however, the underlying mechanisms require further study. RsSOD can be considered as a potential anti-irradiation drug for treating irradiation-induced corneal-injury-related diseases.

## 5. Conclusions

In conclusion, a heat-resistant MnSOD derived from the radiation-resistant bacterium *Radiobacillus* sp. RsSOD was expressed in *E. coli* and purified through repeated freezing and thawing, heat treatment, and DEAE anion-exchange chromatography. The enzyme activity of RsSOD was measured at 2072.5 U/mg. RsSOD exhibited robust resistance to high temperatures, acid, alkali, and artificial intestinal fluid. Importantly, a low concentration of RsSOD at 6.25 U/mL could significantly promote the proliferation of HCE-T cells and protect them from damage induced by both short-term and long-term UV irradiation. Additionally, the RsSOD protein could reduce the apoptosis caused by short-term UV exposure, highlighting its potential anti-UV irradiation effect. These findings suggest that RsSOD could be a promising candidate for treating radiation-induced ocular diseases.

## Data Availability

The data presented in this study are available upon request from the corresponding authors.

## Abbreviations

SOD	Superoxide dismutase
MnSOD	Manganese superoxide dismutase
RsSOD	*Radiobacillus* sp. SOD
IPTG	Isopropyl β-D-1-thiogalactopyranoside
UV	Ultraviolet
MDA	Maleicdialdehyde
HCE-T	Human corneal epithelial cells-transformed
ROS	Reactive oxygen species
ORF	Open reading frame
mtDNA	Mitochondrial DNA
DEAE	Diethylaminoethyl
WST-8	2-(2-methoxy-4-nitrophenyl)-3-(4-nitrophenyl)-5-(2,4-disulfophenyl)-2H-tetrazolium sodium salt
